# Analysis of the Gut Microbiota and Inflammatory Factors in mGluR5-Knockout Mice

**DOI:** 10.3389/fpsyt.2020.00335

**Published:** 2020-04-30

**Authors:** Guohong Cai, Yuanyuan Zhu, Jing Chen, Suo Zhao, Liying Wang, Mengmeng Wang, Jing Huang, Shengxi Wu

**Affiliations:** ^1^Department of Neurobiology, School of Basic Medicine, Fourth Military Medical University, Xi'an, China; ^2^Department of Anatomy, School of Basic Medicine, Fourth Military Medical University, Xi'an, China

**Keywords:** depression, gut microbiota, inflammation, mGluR5, prefrontal cortex

## Abstract

**Introduction:**

Accumulating evidence indicates that the glutamatergic system plays an important role in the development of depression. Notably, the antidepressant effect of metabotropic glutamate receptor 5 (mGluR5) modulation is inconsistent across studies. Here, we attempted to identify the involvement of the gut microbiota and inflammation in mGluR5^−/−^ mice.

**Methods:**

mGluR5^−/−^ mice and their wild-type littermates were used in our study. We used the open field (OF) and elevated plus maze (EPM) tests to assess anxiety-like behaviors, and we used the two-day forced swim test (FST) and tail suspension test (TST) to test despair-like behaviors. 16S rDNA was used to analyze the gut microbiota. Enzyme-linked immunosorbent assays (ELISAs) were used to measure the levels of inflammatory factors. Western blotting was used to detect the levels of various proteins.

**Results:**

mGluR5^−/−^ mice had no significant increase or decrease of despair-like behavior in the absence of stress exposure. However, mGluR5^−/−^ mice exhibited despair-like behaviors following stress exposure. No significant changes in other glutamate receptors or representative synaptic proteins were detected in the prefrontal cortex (PFC) or hippocampus of mGluR5^−/−^ mice. Very similar bacterial groups were observed in mGluR5^−/−^ mice and wild-type controls. In addition, there was no significant difference in the *α*-diversity of the microbiota between mGluR5^−/−^ mice and wild-type controls. The levels of all measured cytokines (IL-1*β*, IL-2, IL-4, IL-6, IL-10, and TNF-*α*) did not change significantly in the PFCs or colons of mGluR5^−/−^ mice.

**Conclusion:**

In conclusion, we deduced that mGluR5^−/−^ mice are susceptible to despair-like behavior. The systemic knockout of mGluR5 did not affect the gut microbiota or inflammatory factors in mice.

## Introduction

In the nervous system of vertebrate, glutamate is the most abundant neurotransmitter (1). The glutamatergic system plays an important role in the development of depression and is an essential target of antidepressant drugs (2). Recently, more attention has been focused on the potential roles of metabotropic glutamate receptors (mGluRs) (3–5). The mGluRs are classified into three groups: group I includes mGluR1 and mGluR5, group II includes mGluR2 and mGluR3, and group III includes mGluR4, mGluR6, mGluR7, and mGluR8 (6, 7). Among them, group I mGluRs are distributed primarily at postsynaptic excitatory synapses and are related to many neuropsychiatric diseases, such as anxiety, stress disorders, neurodegeneration, and depression (3, 5, 8).

It is noticeable, however, that the antidepressant-like effect of mGluR5 modulation is still controversial. Some studies showed that the antagonists of mGluR5 can effectively alleviate depression-like behaviors in rodents (9) and that the basal immobility of mGluR5^−/−^ mice decreased in the forced swim test (FST) (9) and tail suspension test (TST) (1). However, Shin et al.'s study showed that in many stress-induced models of depression, mGluR5^−/−^ mice exhibited increased depression-like behaviors which could be reversed by rescue of mGluR5 in the shell of the nucleus accumbens (NAc) (10).

In addition, many studies have shown that the gut microbiota and inflammation play a major role in the pathophysiological process of depression (11–13). Do gut microbiota and inflammation also affect the mGluR5-related mouse model? Here, we attempted to identify the involvement of the gut microbiota and inflammation in mGluR5^−/−^ mice, a controversial model linked to depression.

## Methods

### Animals

mGluR5^−/−^ mice and wild-type littermates were purchased from Nanjing BioMedical Research Institute of Nanjing University (NBRI). All experimental mice were housed in groups in a room with controllable temperature and humidity and a 12/12-h light/dark cycle. mGluR5^−/−^ mice were divided into three groups, group one (n = 7) for the open field (OF) and FST, group two (n = 7) for elevated plus maze EPM and TST, and group three (n = 8) for gut microbiota, protein, and inflammatory factor analysis.

### Behavioral Testing

#### Open Field Test

The experimental process is as described in the previous study (14). Briefly, mice were tested in a white plastic box measuring 40 cm × 40 cm × 40 cm under full-light conditions (1,000 lux). At the beginning of the experiment, the mouse was placed in the center of the arena. The duration of each video recording was 5 min. Mice were taken to their home cages after video recording. We used an automated analysis system (SMART 3.0, Panlab S.L.U.) to analyze the time spent in the center of the arena, which was used to evaluate anxiety levels.

#### Elevated Plus Maze Test

The experimental process is as described in the previous study (14). Briefly, mice were tested in the black plastic equipment with four arms measuring 50 cm × 5 cm each, which were rested on a platform 1 m from the ground. There are two closed arms with 15-cm-high walls and two open arms without walls. At the beginning of the experiment, the mouse was placed in the center of a platform facing one of the open arm. The duration of each video recording was 5 min, after which the mouse was taken to its home cage. We used an automated analysis system (SMART 3.0, Panlab S.L.U.) to analyze the time spent in the open arms, which was used to evaluate anxiety levels.

#### Two-Day Forced Swim Test

The experimental process is as described in the previous study (14). Briefly, mice were tested in a glass cylinder with 24°C water. The duration of each video recording was 6 min, and afterwards the mouse was taken from the tank into an individual cage for recovery for 90 min. The last 4 min of the FST video was used to assess immobility of the mouse. Twenty-four hours later, the mice were repeatedly tested under the same conditions.

#### Two-Day Tail Suspension Test

The experimental process is as described in the previous study (15). Briefly, mice were suspended 50 cm above the floor acoustically and visually isolated by adhesive tape, which was placed one-third of the way from the tip of the tail. The last 4 min of the 6-min period was used to analyze the immobility time. Mice were suspended by the tail on 2 consecutive days.

### Gut Microbiota Analysis

#### DNA Extraction and Detection

The fecal samples were collected before behavioral testing, placed in 1.5 ml tubes and stored at −80°C. For mGluR5^−/−^ mice and wild-type mice, fecal samples were collected before behavioral testing. The genomic DNA of each sample was extracted by Beijing Genomics Institute Tech Solutions Co., Ltd. (Shenzhen, Guangdong, China). The microplate reader and agarose gel electrophoresis were used to analyze DNA concentration and integrity.

#### 16S rDNA Compositional Sequencing

The 16S rDNA compositional sequencing process is as described in the previous study (16). Once the DNA sample was received, a quality test was performed first, and then a library was constructed of all the qualified DNAs. The T4 DNA polymerase, Klenow fragment, and T4 polynucleotide kinase were used to convert the jagged ends of PCR products into blunt ends. Then, we added an ‘A' base to each 3′ end to make it easier to add adapters. We used fusion primers with dual indexes and adapters for PCR. In both cases, we only used the qualified libraries for sequencing, and the next bioinformatic analysis was based on the results of the sequencing.

#### Bioinformatic Analysis

The raw data were filtered to obtain clean reads by eliminating adapter pollution and low-quality reads, and then paired-end reads with overlaps were merged to tags, which were clustered into OTUs (operational taxonomic units) at 97% sequence similarity (17). The Ribosomal Database Project (RDP) Naïve Bayesian Classifier v.2.2. was used to assign taxonomic ranks to OTU-representative sequences. The tag numbers of each taxonomic rank or OTU in different samples were summarized in a profiling table or histogram, which was drawn with the software R (v3.1.1) (18). The different species screenings and *α*-diversity were analyzed based on OTU and taxonomic ranks.

### Brain and Colon Tissue Cytokine Detections

Brain (prefrontal cortex, PFC) and colon tissue of mGluR5^−/−^ mice and wild-type littermates (group three) were collected. Samples were analyzed using the MSD V-Plex Custom Mouse Cytokine kit (4A Biotech Co., Ltd, China) per the vendor instructions.

### Western Blot Analysis

The experimental process is as described in the previous study (19). PFC and hippocampal tissues of mGluR5^−/−^ mice and wild-type littermates (group three) were collected and lysed in 100–300 μl of Radio Immunopreciptation Assay (RIPA) lysis buffer (10 mM Tris, 1 mM EDTA, 0.5% NP-40, 150 mM NaCl, and 1% Triton X-100 at pH 7.4). The RIPA lysis buffer contained a 1:100 (v/v) ratio of a protease inhibitor cocktail and a phosphatase inhibitor cocktail (Roche). The BCA protein assay (Pierce) was used to quantify the total protein samples (20–40 μg), and then the samples were resolved *via* sodium dodecyl sulfate polyacrylamide gel electrophoresis (SDS-PAGE) and transferred to PVDF membranes. The primary antibodies were as follows: anti-*β*-actin (1:1,000, Cell Signaling Technology); anti-mGluR5 (1:1,000, Abcam); anti-NR2A (1:1,000, Cell Signaling Technology); anti-NR2B (1:1,000, Cell Signaling Technology); anti-PSD95 (1:1,000, Abcam); anti-homer1 (1:1,000, Abcam); and anti-Erk1/2 (1:1,000, Cell Signaling Technology). The enhanced chemiluminescence (ECL) detection method (Advansta) was used to visualize all Western blots. Image J software (version 1.47) was used to quantify the scanned images.

### Statistical Analyses

All data except for data on the microbiota are expressed as means ± SEM. The unpaired t-test, two-sided t-test, or one- or two-way ANOVA was used to test the statistical significance according to the experimental design. The significant differences in microbiota compositions were assessed by the Mann–Whitney U test. Multiple comparisons were corrected by *P*-values using the Benjamini–Hochberg (BH) correction [false discovery rate (FDR) < 0.05].

## Results

### mGluR5^−/−^ Mice Exhibited Despair-Like Behaviors After Stress Exposure

**Figure 1:**
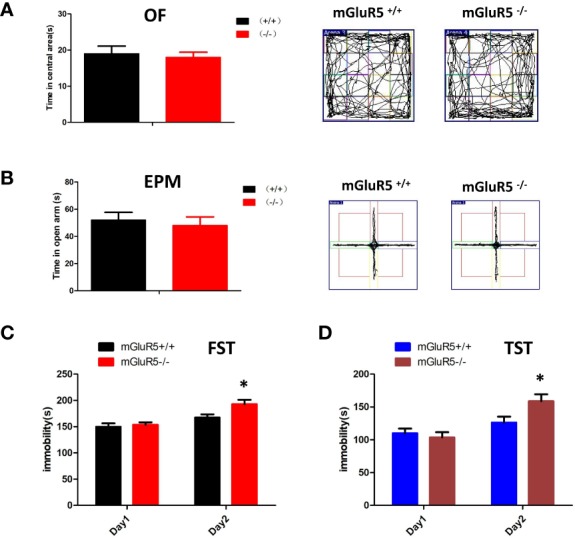
**(A, B)** mGluR5^−/−^ mice exhibit despair-like behaviors after stress exposure. **(A)** Anxiety-like behaviors assessed in OF tests. **(B)** Anxiety-like behaviors assessed in EPM tests. **(C)** The immobility in FST was measured during the last 4 min of the 6-min video. Two-sided t-test, *P < 0.05. **(D)** The immobility time of TST was measured during the last 4 min of the 6-min video. Two-sided t-test, *P < 0.05. n = 7 mice for mGluR5^+/+^ and 7 for mGluR5**^−^**^/^**^−^**.

Depression is often accompanied by anxiety-like symptoms, therefore, we tested for anxiety-like symptoms in mGluR5^−/−^ mice using the OF test and EPM test. The time spent in the central area of the OF test and the open arms of the EPM test made no significant difference between mGluR5^−/−^ mice and wild-type mice (**Figures 1A, B**).

Although antidepression-like symptoms of mGluR5^−/−^ mice were found by using FST in a previous study (9), our results did not show any difference between wild-type control mice and mGluR5^−/−^ mice in a one-day FST or TST. We then performed two-day FST to test depressive behaviors. Under the two-day FST and TST, wild-type mice and mGluR5^−/−^ mice showed no substantial differences in immobility time on day 1, whereas on day 2, mGluR5^−/−^ mice spent more time immobile than wild-type mice (**Figures 1C, D**). These results suggest that after the first day of stress stimulation, the mice exhibit despair-like symptoms the next day.

### mGluR5^−/−^ Mice Showed No Changes in Other Glutamate Receptors or Synaptic Proteins

**Figure 2:**
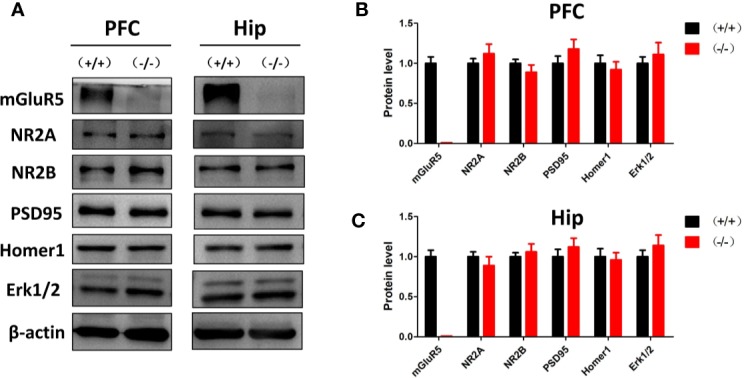
**(A)** Western blot analysis of the prefrontal cortex and hippocampus of mGluR5^+/+^ and mGluR5^−/−^ mice. **(B, C)** mGluR5**^−^**^/^**^−^** mice showed normal levels of NR2A, NR2B, PSD-95, homer1 and extracellular regulated protein kinases (Erk1/2) in the prefrontal cortex and hippocampus. The experiment was successfully repeated three times.

Previous studies suggest that mGluR5 in the PFC and hippocampus may play key roles in the pathological process of depression (20). In the Western blot analysis, mGluR5^−/−^ mice showed normal levels of synaptic scaffold proteins (homer1 and PSD-95), ionotropic glutamate receptors (NR2A and NR2B), and extracellular regulated protein kinases (Erk1/2) in the PFC and hippocampus (**Figure 2**).

### Knockout of mGluR5 Did Not Change the Gut Microbiota

For the controversial mGluR5^−/−^ model, the detection of intestinal microflora may be used to identify the key issues underlying the disease. Therefore, we examined the intestinal microflora of mGluR5^−/−^ mice before stress stimulation. MiSeq sequencing was a total of 1,970,656 raw reads, ranging from 188,224 to 205,284. Paired-end reads were spliced into tags based on the overlap between reads. There were a total of 822,680 tags among all samples, with an average of 82,268 samples and an SD of 705. The stitched tags were optimized to cluster them into OTUs for species classification at 97% similarity. The abundance of the OTUs preliminarily illustrated the species richness of the sample. A total of 476 OTUs were generated from the 10 samples, which were assigned to the taxa from the phylum level to the genus level. The OTU statistics for each sample are shown in **Supplementary Table 1** and **Supplementary Figure 1**.

**Figure 3:**
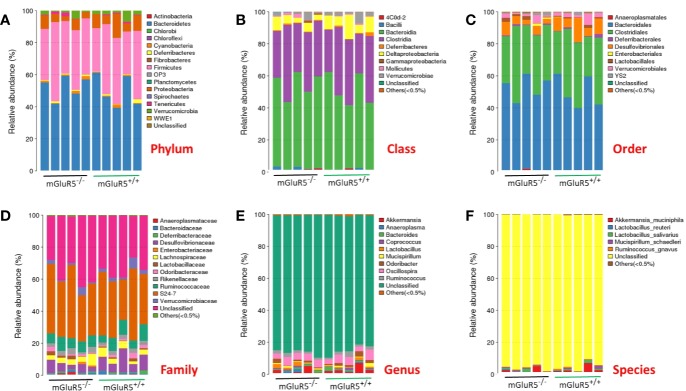
Composition of the gut microbiota at different taxonomic levels. The gut microbiota did not change significantly in mGluR5^−/−^ mice. The taxonomic-composition distribution histograms of each sample are shown at the phylum **(A)**, class **(B)**, order **(C)**, family **(D)**, genus **(E)**, and species **(F)** levels separately. The ratio of each species in a certain sample is displayed by the histogram. The species whose abundances were less than 0.5% in all samples were classified into ‘others' in sub-phylum ranks.

The taxonomic-composition distribution histograms of each sample are shown at the phylum, class, order, family, genus, and species levels separately. The ratio of the species in each sample is displayed using the histograms. No significant changes of taxonomic-composition distribution in the bacterial groups were observed in the mGluR5^−/−^ mice (**Figure 3**).

**Figure 4:**
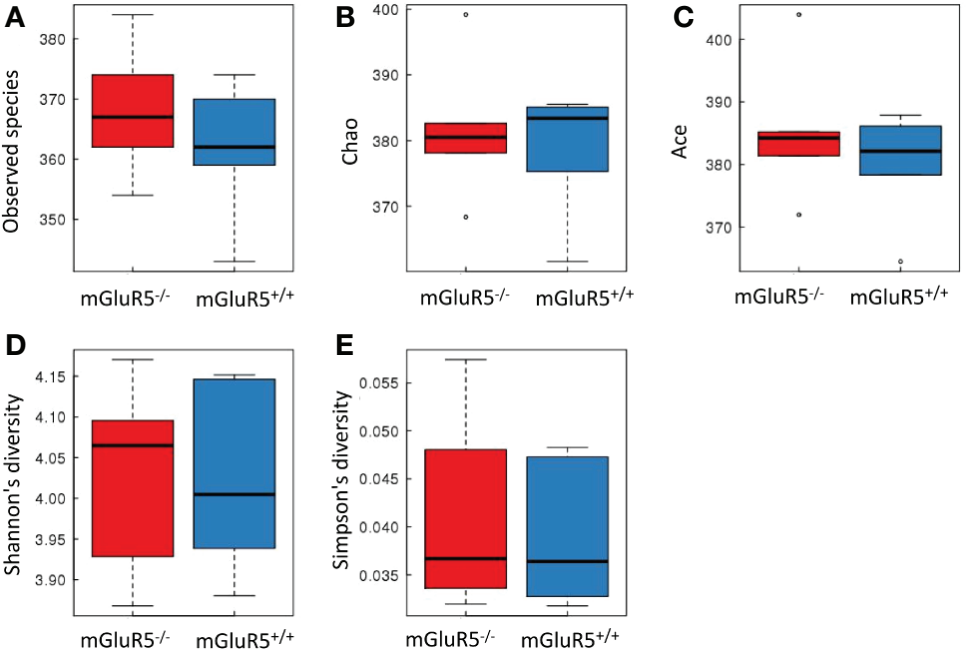
There was no significant difference in the *α*-diversity of microbiota between groups. Bacterial *α*-diversity was tested by observed species **(A)**, Chao **(B)**, Ace **(C)**, Shannon **(D)**, and Simpson indices **(E)**. The Wilcoxon rank-sum test was used for comparisons between two groups. The boxplot of *α*-diversity was drawn, and the analysis above was performed using R software (v3.1.1).

*α*-Diversity is used to analyze the complexity of species diversity by using multiple indices (21), including observed species, Chao, Ace, Shannon and Simpson indices. No significant difference was observed in the *α*-diversity of microbiota between mGluR5^−/−^ mice and wild-type mice (**Figure 4**).

### Cytokines in the PFC Did Not Change Significantly in mGluR5^−/−^ Mice

**Figure 5:**
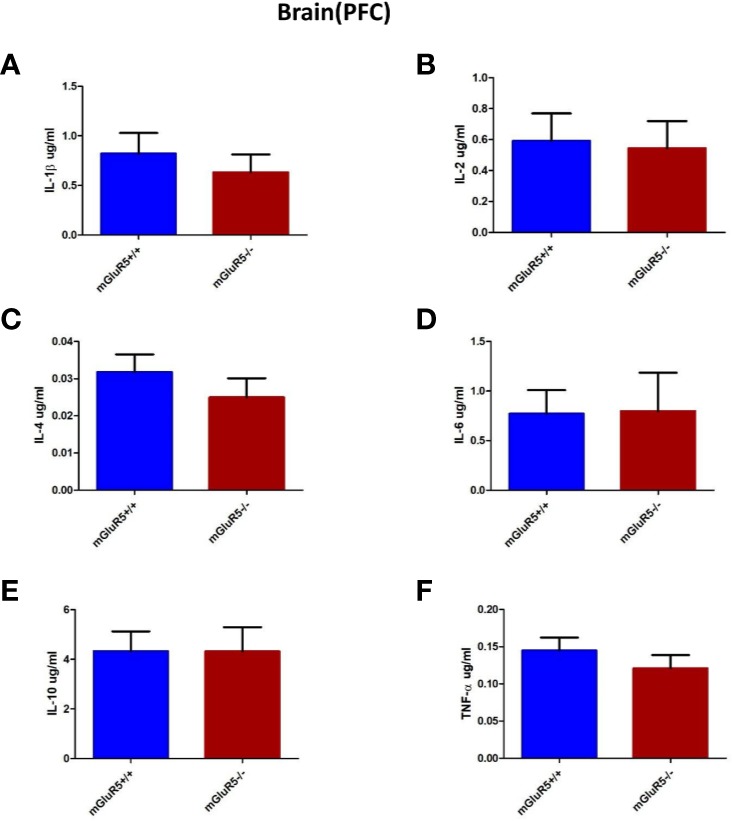
Brain (PFC) cytokine analysis in two groups of mice (mGluR5^+/+^ and mGluR5^−/−^). The levels of all IL-1*β* **(A)**, IL-2 **(B)**, IL-4 **(C)**, IL-6 **(D)**, IL-10 **(E)**, and TNF-α **(F)** did not change significantly in the PFC of mGluR5^−/−^ mice.

Cytokines in the PFC, including IL-1*β*, IL-2, IL-4, IL-6, IL-10, and TNF-*α*, were assessed as markers of inflammation. The levels of these cytokines did not change significantly in the PFC of mGluR5^−/−^ mice (**Figures 5A–F**).

### Cytokines in the Colon Did Not Change Significantly in mGluR5^−/−^ Mice

**Figure 6:**
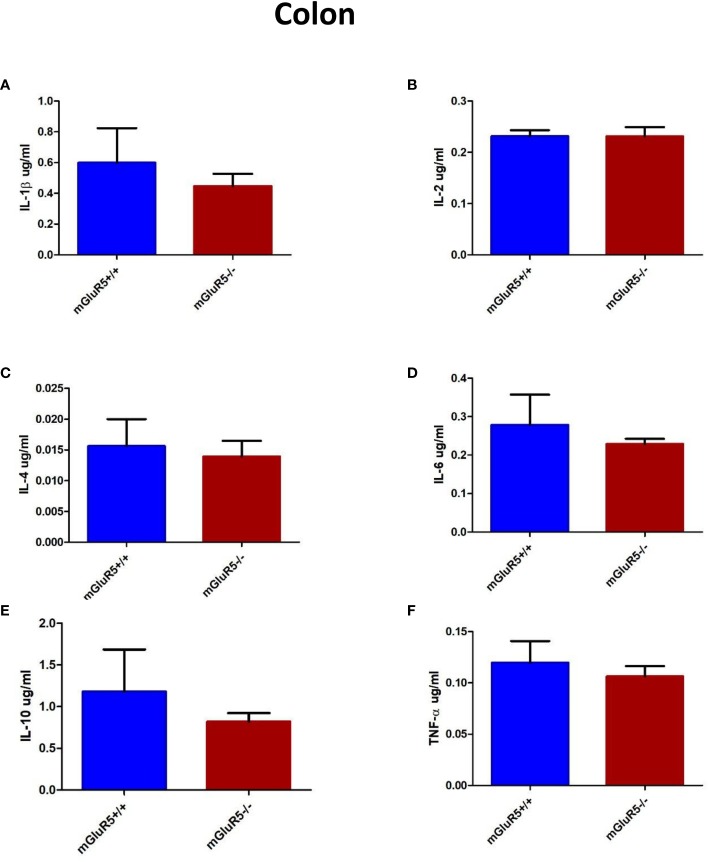
Colon cytokine analysis in two groups of mice (mGluR5^+/+^ and mGluR5^−/−^). The levels of all IL-1*β* **(A)**, IL-2 **(B)**, IL-4 **(C)**, IL-6 **(D)**, IL-10 **(E)**, and TNF-*α* **(F)** did not change significantly in the colons of mGluR5^−/−^ mice.

Colonic levels of inflammatory cytokines play a key role in some patients with depression (22). In our study, the levels of cytokines in the colon, including IL-1*β*, IL-2, IL-4, IL-6, IL-10, and TNF-*α* were assessed as markers of inflammation. The levels of these cytokines did not change significantly in the colons of mGluR5^−/−^ mice (**Figures 6A–F**).

## Discussion

The mGluR5^−/−^ mouse is a promising genetic tool for studying psychiatric diseases. Our results suggest that mGluR5^−/−^ mice had no significant despair-like or antidepressant behavior in the absence of stress exposure. Furthermore, there was no significant change in the intestinal flora and the levels of inflammatory factors in the PFC and colon. Notably, mGluR5^−/−^ mice exhibited despair-like behaviors following stress exposure.

Previous studies have indicated that mGluR5 was involved in many neuropsychiatric disorders (4, 5). For example, Wijetunge et al. used mGluR5^−/−^ mice to research the role of mGluR5 in pattern formation (23). Carvalho et al. demonstrated that the cortex and striatum of mGluR5^−/−^ mice showed reduced number of neurons at 12 months of age and provided evidence that mGluR5 plays an important role in brain aging through modulating multiple cell types (8). Most recently, a study showed that mGluR5^−/−^ mice have some translationally relevant abnormalities associated with schizophrenia (24).

In fact, the depression-related behaviors of mGluR5^−/−^ mice are still controversial. In 2006, Li et al. demonstrated for the first time that the mGluR5-knockout mouse exhibits antidepressant-like behavior (9). Subsequently, Chen et al. found that mGluR5^−/−^ mice had increased number of spine densities, which may partly explain the hyperexcitability observed in mGluR5^−/−^ mice (25). Recently, Liu et al. verified the antidepressant-like effects of mGluR5 in whole-body knockout mice. In addition, transplanting bone marrow from mGluR5^−/−^ mice to wild-type mice can also induce depression-like behaviors (1).

However, in the present study, we did not detect significant depression-like or antidepressant behavior in mGluR5^−/−^ mice, which is similar to the previous study by Shin et al. (10). There are several possible reasons why our findings are inconsistent with previous findings. First, mGluR5 KO mice were constructed differently. For example, the congenic global mGluR5 KO mice used by Liu et al. were based on embryonic stem cell gene targeting technology (1). In our study, the Grm5^flox/flox^ mice were crossed with B6.C-Tg (CMV-cre) mice (NBRI, China) to generate the mGluR5 KO mice. Second, the differences in the process of despair-like behavioral tests may also affect the results. For example, every test session of the TST in Liu et al.'s study lasted for 5 min, and the whole period was scored for immobility (1). In our study and Shin et al.'s study (10), the immobility time of TST was analyzed during the last 4 min of the 6-min period.

In addition to analyzing behavior, we analyzed the gut microbiota, which plays a major role in the pathophysiological processes of depression (11–13). The gut microbiota in stress-induced depression models changes significantly (26–29). However, no study has been conducted to observe changes in the gut microbiota in mGluR5 KO mice. Consistent with our behavioral results, we did not detect abnormalities in the gut microbiota. A recent study showed that there is no significant correlation between gut microbiome and genetic ancestry, and that host genetics play a secondary role in determining microbiome composition (30). Over 20% of the interpersonal microbiome variability is related to factors associated with drugs, diet, and anthropometric measurements (30). Our results further confirm that the effect of host gene changes on the intestinal microflora may be less pronounced than that of the external stimuli.

Accumulating evidence suggests that inflammation is involved in depression (11, 31). A meta-analysis published in 2010 showed significantly higher levels of proinflammatory cytokines, such as IL-6 and TNF-*α*, in depressed groups than in control groups (32). In addition, a more recent systematic review concluded that antidepressant treatment significantly decreases IL-6 and TNF-*α* levels (31). Interestingly, Norman et al. showed that the expression of IL-1*β* gene increased in the frontal cortex of a depression mouse model and that depression-like behavior is blocked by the injection of an IL-1 receptor antagonist (33, 34), which implicates neuroinflammatory activity in the brain as an underlying mechanism of depression. In the present study, we measured the cytokines in the PFC and colon. The results showed that the levels of the measured cytokines (IL-1*β*, IL-2, IL-4, IL-6, IL-10, and TNF-*α*) did not change in mGluR5^−/−^ mice, which further suggests the reliability of our behavioral results.

In one previous study, Shin et al. found that mGluR5^−/−^ mice exhibited depression-like behaviors after stress exposure, which can be rescued by increasing mGluR5 expression in the nucleus accumbens (10). The limitations of FST and TST for detecting the resilience state have been documented by several studies using specific gene knockout animal models (10, 35). In our study, the 2 d-FST and 2 d-TST were used instead of the 1 d-FST and 1 d-TST. Consistent with published results, mGluR5^−/−^ mice exhibited despair-like behavioral phenotype after stress exposure in the present study. Although mGluR5^−/−^ mice showed no difference in the expression levels of NR2A, NR2B, PSD-95, homer1 and Erk1/2 in the PFC and hippocampus, the systemic knockout of mGluR5 still caused problems in mood regulation. The mechanism that specifically leads to enhanced susceptibility to despair-like behaviors requires more research.

Our research had some limitations. First, despair-like behavior testing in mice is not comprehensive, and there are many detection methods that have not been applied, such as the novelty-suppressed feeding test, sucrose preference test, nesting test and splash test, *etc*. Second, research on inflammation levels in mGluR5^−/−^ mice requires evidence from other tissues. Third, the sample size of our study was small, and the conclusion we draw needs to be verified by future studies.

In conclusion, we deduced that mGluR5^−/−^ mice are susceptible to despair-like behavior. The systemic knockout of mGluR5 did not affect the gut microbiota or inflammatory factors in mice.

## Data Availability Statement

The datasets generated for this study can be found in the BioProject ID : PRJNA605506.

## Ethics Statement

All experimental procedures were approved by the Institutional Animal Care and Use Committee (IACUC) at the Fourth Military Medical University and conformed to the Guide for the Care and Use of Laboratory Animals published by the National Institutes of Health (NIH). All efforts were made to minimize animal suffering and to reduce the number of animals that were used.

## Author Contributions

SW conceived and designed the experiments. GC and YZ performed most of the experiments and analyzed the data. GC and JC wrote and refined the article. MW participated in the animal modeling and behavioral experiments. LW and SZ assisted in laboratory work and figure preparation. JH supervised the acquisition of results.

## Funding

This study was supported by the National Natural Science Foundation of China (81730035, 81371240, SW; 81900489, YZ; 31671247, JH; 81401003 JC), and Scientific Research and Sharing Platform Construction Project of Shaanxi Province (2016FWPT-04, SW).

## Conflict of Interest

The authors declare that the research was conducted in the absence of any commercial or financial relationships that could be construed as a potential conflict of interest.

## References

[1] Liu YW Zhao L Zhou M Wang H Yang N Dai SS Transplantation with mGluR5 deficiency bone marrow displays antidepressant-like effect in C57BL/6J mice. Brain Behav Immun (2019) 79:114–24. 10.1016/j.bbi.2019.01.022 30682501

[2] Kugaya A Sanacora G Beyond monoamines: glutamatergic function in mood disorders. CNS Spectr (2005) 10:808–19. 10.1017/s1092852900010403 16400244

[3] Fuxe K Borroto-Escuela DO Basimglurant for treatment of major depressive disorder: a novel negative allosteric modulator of metabotropic glutamate receptor 5. Expert Opin Invest Drugs (2015) 24:1247–60. 10.1517/13543784.2015.1074175 26219727

[4] Pillai RL Tipre DN Metabotropic glutamate receptor 5 - a promising target in drug development and neuroimaging. Eur J Nucl Med Mol Imaging (2016) 43:1151–70. 10.1007/s00259-015-3301-5 26743895

[5] Swanson CJ Bures M Johnson MP Linden AM Monn JA Schoepp DD Metabotropic glutamate receptors as novel targets for anxiety and stress disorders. Nat Rev Drug Discovery (2005) 4:131–44. 10.1038/nrd1630 15665858

[6] Schoepp DD Conn PJ Metabotropic glutamate receptors in brain function and pathology. Trends Pharmacol Sci (1993) 14:13–20. 10.1016/0165-6147(93)90107-u 7680175

[7] Conn PJ Pin JP Pharmacology and functions of metabotropic glutamate receptors. Annu Rev Pharmacol Toxicol (1997) 37:205–37. 10.1146/annurev.pharmtox.37.1.205 9131252

[8] Carvalho TG Alves-Silva J de Souza JM Real A Doria JG Vieira E Metabotropic glutamate receptor 5 ablation accelerates age-related neurodegeneration and neuroinflammation. Neurochem Int (2019) 126:218–28. 10.1016/j.neuint.2019.03.020 30930274

[9] Li X Need AB Baez M Witkin JM Metabotropic glutamate 5 receptor antagonism is associated with antidepressant-like effects in mice. J Pharmacol Exp Ther (2006) 319:254–9. 10.1124/jpet.106.103143 16803860

[10] Shin S Kwon O Kang JI Kwon S Oh S Choi J mGluR5 in the nucleus accumbens is critical for promoting resilience to chronic stress. Nat Neurosci (2015) 18:1017–24. 10.1038/nn.4028 26005851

[11] Dantzer R O'Connor JC Freund GG Johnson RW Kelley KW From inflammation to sickness and depression: when the immune system subjugates the brain. Nat Rev Neurosci (2008) 9:46–56. 10.1038/nrn2297 18073775 PMC2919277

[12] Wohleb ES Franklin T Iwata M Duman RS Integrating neuroimmune systems in the neurobiology of depression. Nat Rev Neurosci (2016) 17:497–511. 10.1038/nrn.2016.69 27277867

[13] Flux MC Lowry CA Finding intestinal fortitude: Integrating the microbiome into a holistic view of depression mechanisms, treatment, and resilience. Neurobiol Dis (2020) 135:104578. 10.1016/j.nbd.2019.104578 31454550 PMC6995775

[14] Scott KA Ida M Peterson VL Prenderville JA Moloney GM Izumo T Revisiting Metchnikoff: Age-related alterations in microbiota-gut-brain axis in the mouse. Brain Behav Immun (2017) 65:20–32. 10.1016/j.bbi.2017.02.004 28179108

[15] Kim Y Lee HY Cho SH Antidepressant Effects of Pharmacopuncture on Behavior and Brain-Derived Neurotrophic Factor (BDNF) Expression in Chronic Stress Model of Mice. J Acupunct Meridian Stud (2017) 10:402–8. 10.1016/j.jams.2017.08.007 29275796

[16] Chen D Chen G Wan P Hu B Chen L Ou S Digestion under saliva, simulated gastric and small intestinal conditions and fermentation *in vitro* of polysaccharides from the flowers of Camellia sinensis induced by human gut microbiota. Food Funct (2017) 8:4619–29. 10.1039/c7fo01024a 29143827

[17] Tan L Zhao S Zhu W Wu L Li J Shen M The Akkermansia muciniphila is a gut microbiota signature in psoriasis. Exp Dermatol (2018) 27:144–9. 10.1111/exd.13463 29130553

[18] Liu W Zhang R Shu R Yu J Li H Long H Study of the Relationship between Microbiome and Colorectal Cancer Susceptibility Using 16SrRNA Sequencing. BioMed Res Int (2020) 2020:7828392. 10.1155/2020/7828392 32083132 PMC7011317

[19] Guo B Wang J Yao H Ren K Chen J Yang J Chronic Inflammatory Pain Impairs mGluR5-Mediated Depolarization-Induced Suppression of Excitation in the Anterior Cingulate Cortex. Cereb Cortex (2018) 28:2118–30. 10.1093/cercor/bhx117 28520841

[20] Millard SJ Lum JS Fernandez F Weston-Green K Newell KA Perinatal exposure to fluoxetine increases anxiety- and depressive-like behaviours and alters glutamatergic markers in the prefrontal cortex and hippocampus of male adolescent rats: A comparison between Sprague-Dawley rats and the Wistar-Kyoto rat model of depression. J Psychopharmacol (2019) 33:230–43. 10.1177/0269881118822141 30698051

[21] Schloss PD Westcott SL Ryabin T Hall JR Hartmann M Hollister EB Introducing mothur: open-source, platform-independent, community-supported software for describing and comparing microbial communities. Appl Environ Microbiol (2009) 75:7537–41. 10.1128/AEM.01541-09 PMC2786419 19801464

[22] Abautret-Daly A Dempsey E Parra-Blanco A Medina C Harkin A Gut-brain actions underlying comorbid anxiety and depression associated with inflammatory bowel disease. Acta Neuropsychiatr (2018) 30:275–96. 10.1017/neu.2017.3 28270247

[23] Wijetunge LS Till SM Gillingwater TH Ingham CA Kind PC mGluR5 regulates glutamate-dependent development of the mouse somatosensory cortex. J Neurosci (2008) 28:13028–37. 10.1523/JNEUROSCI.2600-08.2008 PMC3844741 19052194

[24] Aguilar DD Strecker RE Basheer R McNally JM Alterations in sleep, sleep spindle, and EEG power in mGluR5 knockout mice. J Neurophysiol (2020) 123:22–33. 10.1152/jn.00532.2019 31747354 PMC6985862

[25] Chen CC Lu HC Brumberg JC mGluR5 knockout mice display increased dendritic spine densities. Neurosci Lett (2012) 524:65–8. 10.1016/j.neulet.2012.07.014 PMC3727626 22819970

[26] Zhang JC Yao W Dong C Yang C Ren Q Ma M Blockade of interleukin-6 receptor in the periphery promotes rapid and sustained antidepressant actions: a possible role of gut-microbiota-brain axis. Transl Psychiatry (2017) 7:e1138. 10.1038/tp.2017.112 28556833 PMC5534942

[27] Yang C Qu Y Fujita Y Ren Q Ma M Dong C Possible role of the gut microbiota-brain axis in the antidepressant effects of (R)-ketamine in a social defeat stress model. Transl Psychiatry (2017) 7:1294. 10.1038/s41398-017-0031-4 29249803 PMC5802627

[28] Szyszkowicz JK Wong A Anisman H Merali Z Audet MC Implications of the gut microbiota in vulnerability to the social avoidance effects of chronic social defeat in male mice. Brain Behav Immun (2017) 66:45–55. 10.1016/j.bbi.2017.06.009 28629758

[29] Bailey MT Dowd SE Galley JD Hufnagle AR Allen RG Lyte M Exposure to a social stressor alters the structure of the intestinal microbiota: implications for stressor-induced immunomodulation. Brain Behav Immun (2011) 25:397–407. 10.1016/j.bbi.2010.10.023 21040780 PMC3039072

[30] Rothschild D Weissbrod O Barkan E Kurilshikov A Korem T Zeevi D Environment dominates over host genetics in shaping human gut microbiota. Nature (2018) 555:210–5. 10.1038/nature25973 29489753

[31] Kohler CA Freitas TH Stubbs B Maes M Solmi M Veronese N Peripheral Alterations in Cytokine and Chemokine Levels After Antidepressant Drug Treatment for Major Depressive Disorder: Systematic Review and Meta-Analysis. Mol Neurobiol (2018) 55:4195–206. 10.1007/s12035-017-0632-1 28612257

[32] Dowlati Y Herrmann N Swardfager W Liu H Sham L Reim EK A meta-analysis of cytokines in major depression. Biol Psychiatry (2010) 67:446–57. 10.1016/j.biopsych.2009.09.033 20015486

[33] Norman GJ Karelina K Zhang N Walton JC Morris JS Devries AC Stress and IL-1beta contribute to the development of depressive-like behavior following peripheral nerve injury. Mol Psychiatry (2010) 15:404–14. 10.1038/mp.2009.91 PMC5214062 19773812

[34] Norman GJ Karelina K Morris JS Zhang N Cochran M Courtney DA Social interaction prevents the development of depressive-like behavior post nerve injury in mice: a potential role for oxytocin. Psychosom Med (2010) 72:519–26. 10.1097/PSY.0b013e3181de8678 20466999

[35] Krishnan V Han MH Graham DL Berton O Renthal W Russo SJ Molecular adaptations underlying susceptibility and resistance to social defeat in brain reward regions. Cell (2007) 131:391–404. 10.1016/j.cell.2007.09.018 17956738

