# Supplementary material for: Analysis of the Gut Microbiota and Inflammatory Factors in mGluR5-Knockout Mice
Source: Front Psychiatry. 2020 Apr 30;11:335. doi: 10.3389/fpsyt.2020.00335 (PMC7203659; doi:10.3389/fpsyt.2020.00335)
Supplement: Supplementary file 1 [file Presentation_1.pptx]

## Slide 1
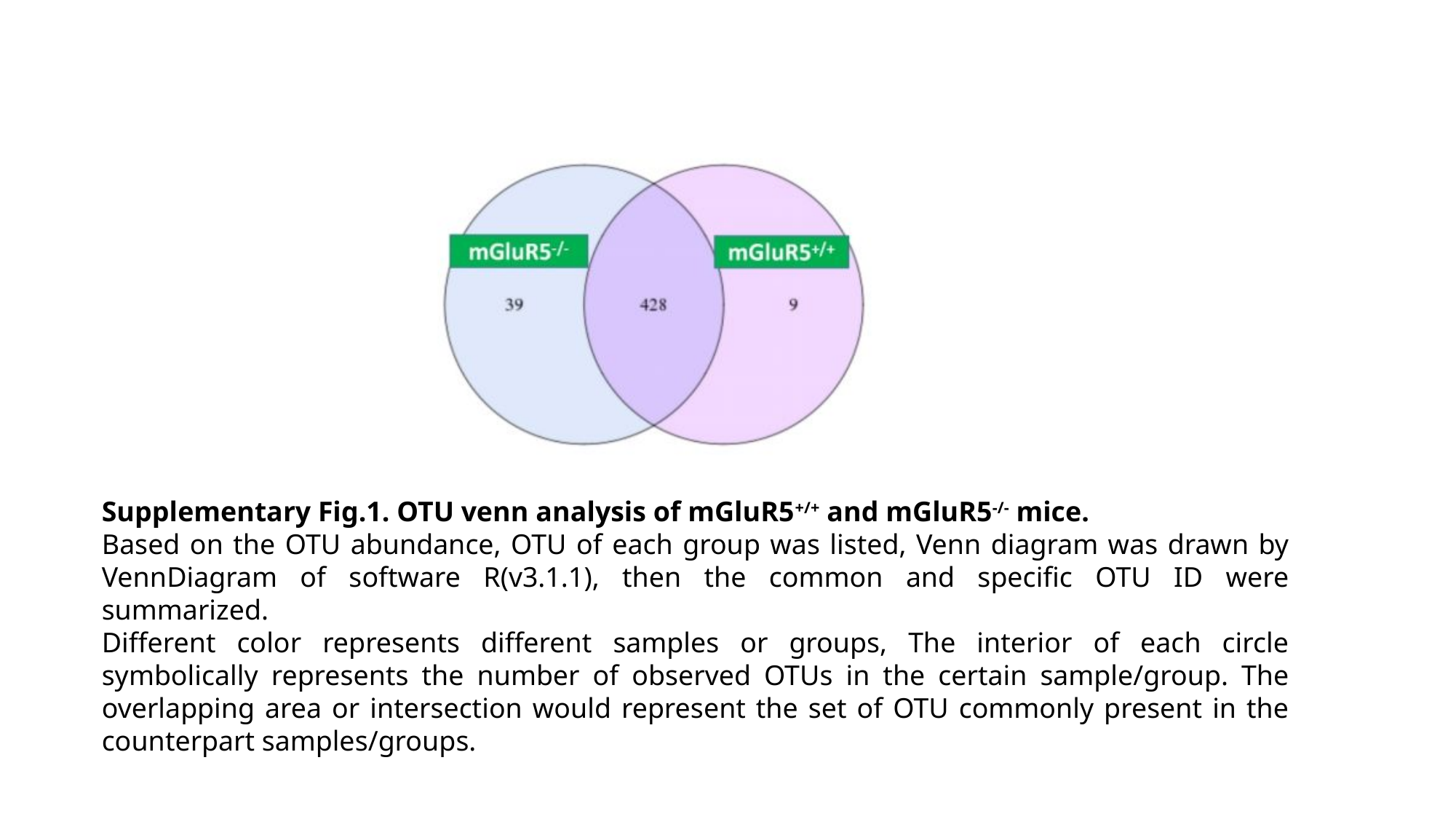

Supplementary Fig.1. OTU venn analysis of mGluR5+/+ and mGluR5-/- mice.
Based on the OTU abundance, OTU of each group was listed, Venn diagram was drawn by VennDiagram of software R(v3.1.1), then the common and specific OTU ID were summarized.
Different color represents different samples or groups, The interior of each circle symbolically represents the number of observed OTUs in the certain sample/group. The overlapping area or intersection would represent the set of OTU commonly present in the counterpart samples/groups.

## Slide 2
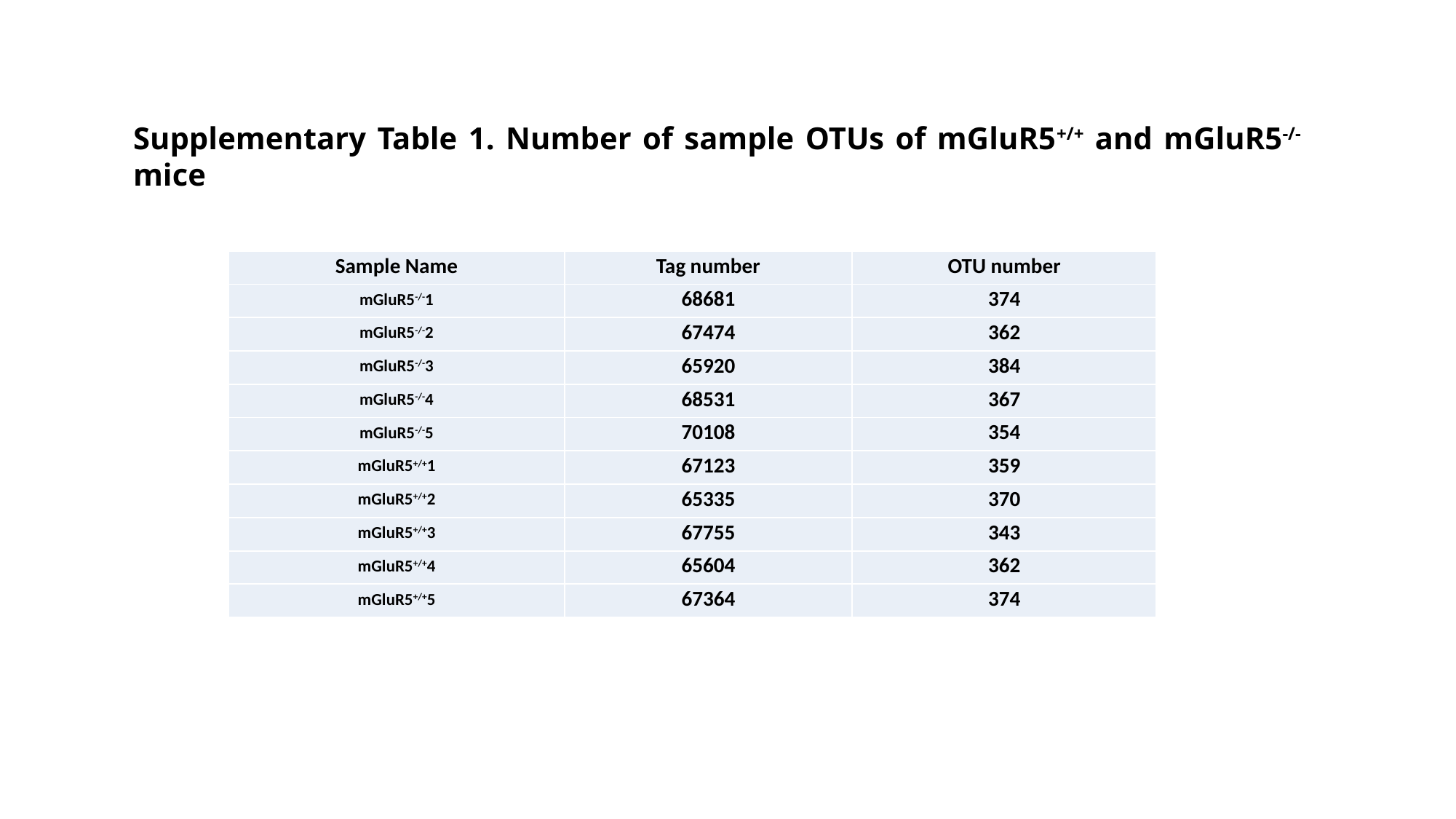

Supplementary Table 1. Number of sample OTUs of mGluR5+/+ and mGluR5-/- mice
| Sample Name | Tag number | OTU number |
| --- | --- | --- |
| mGluR5-/-1 | 68681 | 374 |
| mGluR5-/-2 | 67474 | 362 |
| mGluR5-/-3 | 65920 | 384 |
| mGluR5-/-4 | 68531 | 367 |
| mGluR5-/-5 | 70108 | 354 |
| mGluR5+/+1 | 67123 | 359 |
| mGluR5+/+2 | 65335 | 370 |
| mGluR5+/+3 | 67755 | 343 |
| mGluR5+/+4 | 65604 | 362 |
| mGluR5+/+5 | 67364 | 374 |
